# Development of a nanocomposite ultrafiltration membrane based on polyphenylsulfone blended with graphene oxide

**DOI:** 10.1038/srep41976

**Published:** 2017-02-03

**Authors:** Arun Kumar Shukla, Javed Alam, Mansour Alhoshan, Lawrence Arockiasamy Dass, M. R. Muthumareeswaran

**Affiliations:** 1 King Abdullah Institute for Nanotechnology, King Saud University, P.O. Box- 2455, Riyadh 11451, Kingdom of Saudi Arabia; 2 Chemical Engineering Department, College of Engineering, King Saud University, P.O. Box 800, Riyadh 11421, Kingdom of Saudi Arabia

## Abstract

In the present study, graphene oxide (GO) was incorporated as a nanoadditive into a polyphenylsulfone (PPSU) to develop a PPSU/GO nanocomposite membrane with enhanced antifouling properties. A series of membranes containing different concentrations (0.2, 0.5 and 1.0 wt.%) of GO were fabricated via the phase inversion method, using N-methyl pyrrolidone (NMP) as the solvent, deionized water as the non-solvent, and polyvinylpyrrolidone (PVP) as a pore forming agent. The prepared nanocomposite membranes were characterized using scanning electron microscopy (SEM) and atomic force microscopy (AFM), and were also characterized with respect to contact angle, zeta potential and porosity, mean pore radius, tortuosity and molecular weight cut-off (MWCO). Thermogravimetric analysis (TGA) and tensile testing were used to measure thermal and mechanical properties. The membrane performance was evaluated by volumetric flux and rejection of proteins, and antifouling properties. According to the results, the optimum addition of 0.5 wt% GO resulted in a membrane with an increased flux of 171 ± 3 Lm^−2^h^−1^ with a MWCO of ~40 kDa. In addition, the GO incorporation efficiently inhibited the interaction between proteins and the membrane surface, thereby improving the fouling resistance ability by approximately 58 ± 3%. Also, the resulting membranes showed a significant improvement in mechanical and thermal properties.

Membrane technology has shown great promise in all types of separation such as microfiltration (MF), ultrafiltration (UF), nanofiltration (NF) and reverse osmosis (RO). This technology has a number of attractive features such as simplicity of operating conditions, low energy consumption, no phase change, compact design and environmental friendliness^1^,^2^. Despite its great promise, membrane technology has a key limitation of membrane fouling. This is due to membrane surface properties including hydrophobicity, surface charge and roughness^3^,^4^,^5^,^6^. Fouling results in an increase in the operational cost, as well as decreased membrane life. Therefore, a large part of research in membrane technology is focused on the development of so**-**called antifouling membranes. To date, a number of techniques to develop antifouling membranes have been employed. They include hydrophilic surface modification by coating, grafting and the concept of making thin film composite (TFC) membranes, bulk modification by blending and mixing hydrophilic polymers, and incorporation of nanomaterials into membrane matrices for developing nanocomposite membranes^1^,^7^,^8^,^9^,^10^. Recently, the study of nanocomposite membranes has grown as an active area of membrane materials research and development. The nanomaterials of greatest interest for nanocomposite membrane development are titanium dioxide (TiO_2_), aluminium oxide (Al_2_O_3_), zinc oxide (ZnO), silicon dioxide (SiO_2_), carbon nanotubes (CNT) and graphene oxide (GO)^11^,^12^,^13^,^14^,^15^,^16^,^17^,^18^. Among them, GO is an emerging nanomaterial that has shown great promise in the development of anti-fouling nanocomposite membranes^17^,^19^,^20^,^21^,^22^,^23^. During the past few years, several investigators have studied the incorporation of GO into commonly used polymeric membrane matrices like polysulfone (PSF), polyethersulfone (PES), polyvinylidene difluoride (PVDF), polyacrylonitrile (PAN), and their copolymers, with the aim developing antifouling nanocomposite membranes^19^,^24^,^25^,^26^,^27^. The additional advantages of using GO as nanoadditive is that it can be processed easily owing to the availability of functional groups such as hydroxyl, carbonyl and carboxyl located on its surface and edge, which help to disperse it into the membrane matrix. Apart from these, its high hydrophilicity, antibacterial properties, low toxicity, chemical and biological durability and low cost make GO a very promising nanomaterial for the development of antifouling nanocomposite membranes^17^,^24^,^28^,^29^,^30^,^31^,^32^,^33^,^34^,^35^.

In the present study, GO was incorporated into a polyphenylsulfone, (PPSU) matrix to develop a flat-sheet nanocomposite membrane, hereafter called a PPSU/GO nanocomposite membrane. PPSU, a member of the family of sulfone polymers, is an outstanding material for preparing membranes with superior physical and chemical properties. Its great heat and chemical resistance, and long-term thermal and hydrolytic stability make PPSU a suitable membrane material for any water treatment applications. Despite their advanced features, PPSU membranes exhibit a hydrophobic nature that limits their long term-use in water process systems^36^,^37^. Hence, in this study, GO was mixed with PPSU to prepare a hydrophilic membrane, thereby reducing fouling, leading to enhanced cost-effectiveness by extending the operational lifetime and lowering energy requirements. To the best of our knowledge, GO has not yet been reported to improve the quality of a PPSU membrane, particularly to enhance the hydrophilicity of a PPSU membrane. More specifically, GO has not been investigated as a hydrophilic nanoadditive to prepare a PPSU/GO nanocomposite antifouling membrane. Hence, we prepared such a PPSU/GO nanocomposite membrane and reported the influence of GO addition with three different levels of GO content (0.2, 0.5, and 1.0 wt.%) on the properties of the nanocomposite membrane. The performance of the resultant nanocomposite membrane was evaluated in the terms of volumetric flux and the rejection of two model proteins of different molecular weights (bovine serum albumin BSA and pepsin), as well as its antifouling ability along with the adsorption of protein. The prepared PPSU/GO nanocomposite membrane exhibited superior separation performance compared to the pure PPSU membrane. The 0.5 wt.% GO-based nanocomposite membrane, which showed a flux of 171 ± 3 Lm^−2^h^−1^ and ~95% rejection of proteins, could be used as the preferred nanocomposite membrane in ultrafiltration applications.

## Results and Discussion

### Morphology

**Figure 1:**
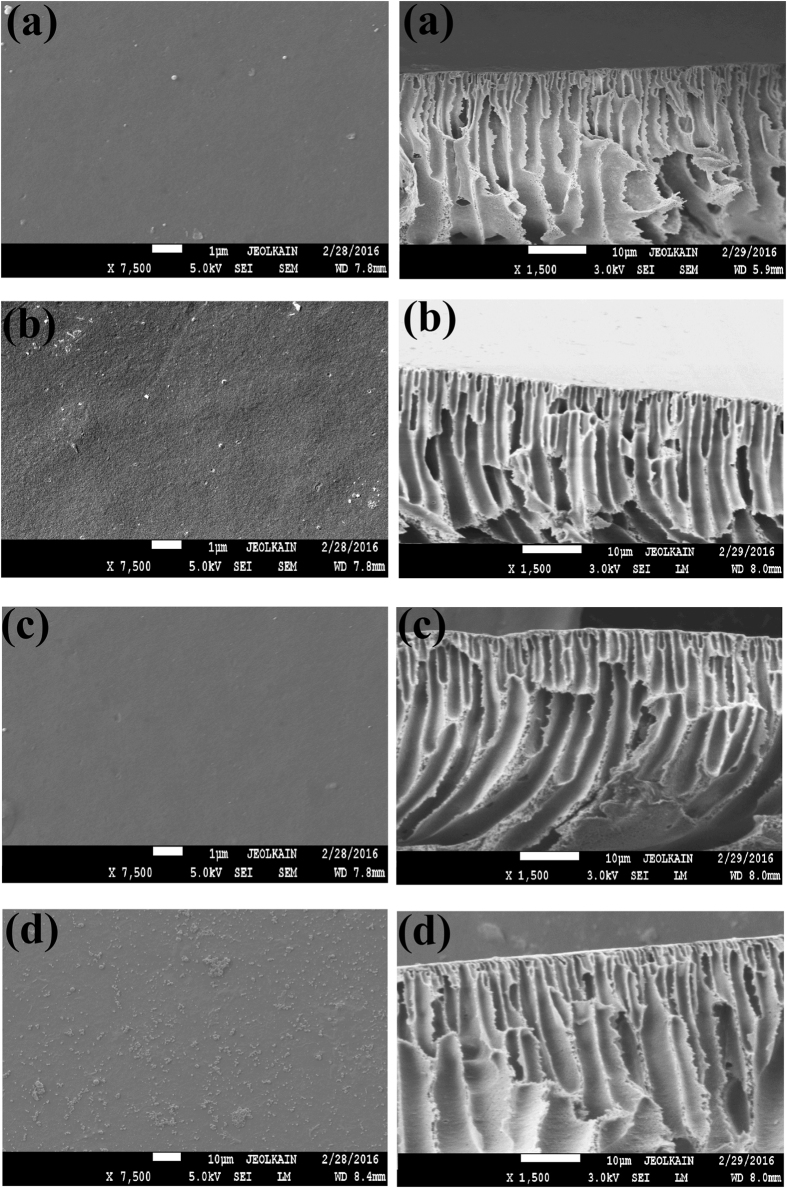
SEM images of membrane top surface and cross-section morphologies. (**a**) PPSU. (**b**) PPSU/GO 0.2 wt.%. (**c**) PPSU/GO 0.5 wt.%. (**d**) PPSU/GO 1.0 wt.%.

**Figure 2:**
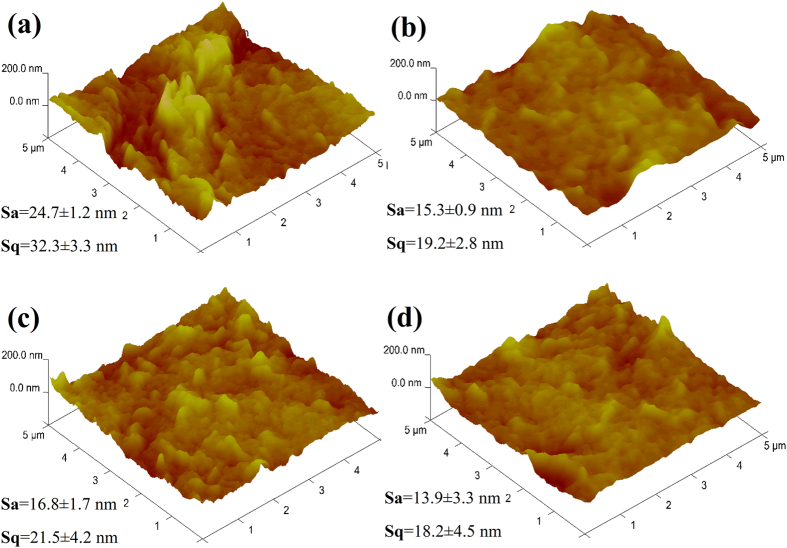
AFM 3D images of the prepared membranes surface. **(a)** PPSU. **(b)** PPSU/GO 0.2 wt.%. **(c)** PPSU/GO 0.5 wt.%. **(d)** PPSU/GO 1.0 wt.%.

Figure 1 shows both surface and cross-section morphologies of all the prepared membrane samples. As shown in the SEM images (Fig. 1a), the pure PPSU membrane had randomly distributed drop-like macro-voids. The walls of the macro-voids are thick and with closed ends. Also, a sponge layer was grown in a significant part of the membrane. Moreover, it was found that a thick skin layer was formed on top of a porous macro-void structure. With the addition of GO, the morphology of the nanocomposite membrane changed in many ways. Firstly, the pores in the sub porous layer of the membrane were relatively thinner and appeared in straight finger structures with open ends. Secondly, the thickness of the skin layer was reduced, and very fine oval-shaped pores were formed just under this thin-skin layer. Lastly, the spongy support layer thickness was also reduced but appeared much denser with interconnected pores compared with that of the PPSU membrane. Apart from these, it was found that the difference in the loading of GO in the PPSU matrix membrane afforded various types of structures. A membrane prepared from 0.2 wt.% GO exhibited small pores, residing underneath the skin layer (Fig. 1b), and the density and length of the pores increased for the membrane prepared from 0.5 wt.% GO (Fig. 1c). The pores stretched from the thin-skin layer to the supporting layer when the GO wt.% increased from 0.5 to 1.0% (Fig. 1d). Hence the membrane morphology, particularly of the porous sublayer, was found to depend on the loading of GO. Typically, this is due to a significant effect of hydrophilicity of the membrane casting solution with the GO concentration. The GO increases the exchange rate between the solvent and non-solvent during the precipitation process. This results in the development of a membrane with highly porous morphology coupled with dense pores and microscopic voids^27^,^38^. It can be seen in Fig. 2 that the surface roughness of the PPSU membrane is lower than the membrane prepared from GO, but among the membranes prepared with three different concentrations of GO (0.2, 0.5, 1.0 wt.%) the membrane with 0.5 wt.% GO showed higher values of roughness parameters. An addition of 0.5 wt.% GO resulted in significant changes in membrane topography features also. The large peaks and valleys were changed to a large number of small peaks and valleys, owing to the good compatibility of GO with the PPSU matrix. This led to a suitable surface structure being developed in the membrane.

### Thermal and mechanical properties

**Figure 3:**
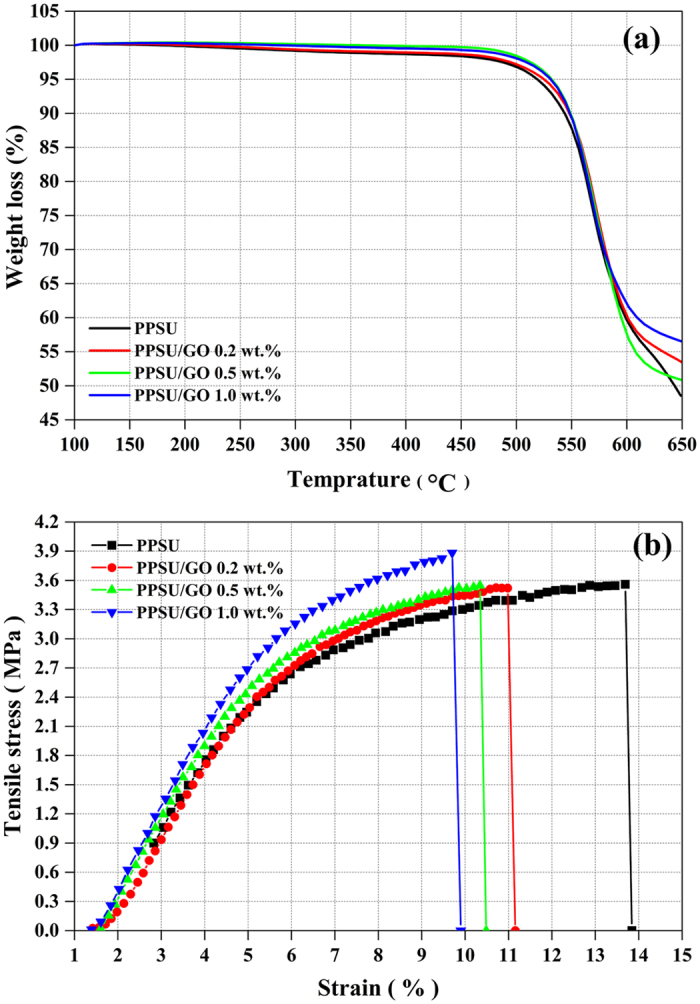
**(a)** Thermal properties and **(b)** mechanical properties using tensile tests of prepared membrane samples.

Figure 3(a) shows the thermal properties of the prepared membranes investigated at a temperature range of 100 °C to 650 °C. Results showed that the addition of GO to the PPSU matrix enhanced the thermal stability of the membrane. An increased thermal stability of the nanocomposite membranes is credited to the availability of the polar functional groups of GO, leading to the strong interfacial bonding between the PPSU matrix and the GO nanoadditive. The thermal properties of membranes with a GO content also have been described by other researchers^29^,^39^,^40^. Figure 3(b) shows the mechanical properties of the prepared membranes determined by the tensile test and defined by stress–strain curves with respect to three different GO contents. As shown in curve, the pure PPSU membrane showed a 3.5 ± 0.1 MPa tensile strength. This value increased to 3.8 ± 0.1 MPa when 1.0 wt.% GO was added. Furthermore, when the GO content increased from 0.2 wt.% to 0.5 no significant difference in tensile strength was observed. Meanwhile, at the higher level loading of GO, the elongation-at-break percentages decreased, reaching to 11.1 ± 0.1% for 0.2 wt.% GO, 10.5 ± 0.1% for 0.5 wt.%, and 9.9 ± 0.2% for 1.0 wt.% GO. The optimal mechanical properties for the membrane prepared from 1.0 wt.% GO can be credited to strong interaction between polymer matrix and GO owing to uniform dispersion of GO^26^,^29^,^31^. The above results were well in agreement with TGA and indicate that the PPSU/GO nanocomposite membranes also have good mechanical stability.

### Hydrophilicity, porosity, mean pore radius and pore tortuosity

**Figure 4:**
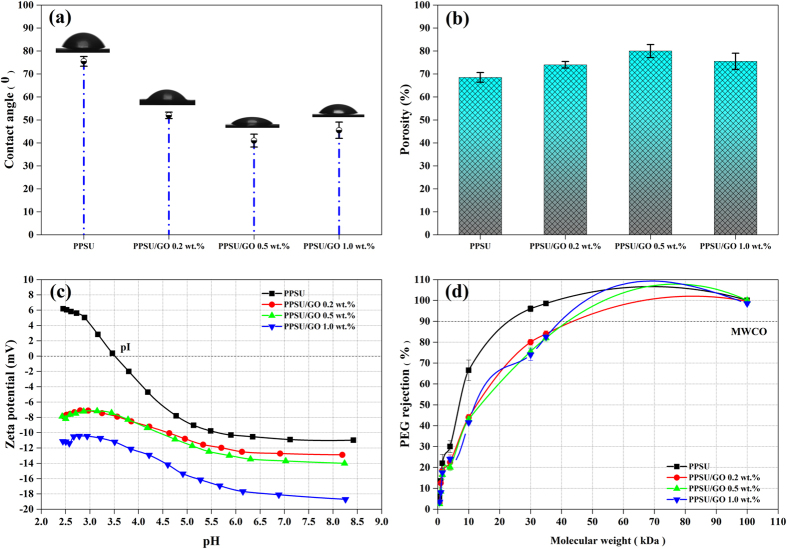
Membrane properties of PPSU, PPSU/GO 0.2 wt.%, PPSU/GO 0.5 wt.% and PPSU/GO 1.0 wt.% membranes. **(a)** Water contact angle. **(b)** Overall porosity determined by the gravimetric method. **(c)** The surface zeta potential as a function of pH. **(d)** The surface pore size in terms of MWCO using a 1 g/L neutral solution of PEG and PEO.

Figure 4(a) shows results of the contact angle analysis for the prepared membranes. As expected, the contact angle values were found to be dependent on the GO wt.%. According to the results, the pure PPSU membrane had a contact angle of 75 ± 2°. This value decreased to 52 ± 1° upon 0.2 wt.% loading of GO. The optimum contact angle of the nanocomposite membrane decreased to 41 ± 2°, when 0.5 wt.% GO was added. However, the contact angle value was found to increase to 45 ± 3° when 1.0 wt.% GO was added; this might be due to GO agglomeration in the PPSU matrix. The decreased contact angle for the nanocomposite membrane prepared with 0.5 wt.% GO was credited to the good dispersion of GO in the PPSU matrix, which generated more oxygenated contained functional groups on the membrane surface^5^,^41^. Similar observations were also reported for the GO in different polymeric matrices^27^,^31^,^42^. Subsequently, membrane porosity and volumetric flux were improved compared to the PPSU membrane, which is discussed in detail in the next section. The effect of GO concentrations on membrane overall porosity, mean pore radius and pore tortuosity can be observed in Fig. 4(b) and Table 1. In our investigation, we observed that when the GO content was 0.2 wt.% and 0.5 wt.%, the porosity initially increased in the range of 74 ± 1.4–80 ± 2.8%, decreasing its pore tortuosity. This can be explained on the basis of the low hydrophilic nature of GO in the PPSU matrix, which increased the solution thermodynamic instability in the phase inversion process and resulted in the formation of large pores on the membrane. When the GO concentration was increased to 1.0 wt.%, the porosity of the membrane was reduced to 75.5 ± 3.5%. This is credited to agglomeration of GO in the PPSU matrix upon higher loading, leading to a decrease in the exchange rate of the solvent and non-solvent (water) during the phase inversion process. Thus, the solvent was not properly leached out, which consequently led to a lower porosity as well as a reduced mean pore radius in the membrane. These phenomena were similar to those reported by other researchers^27^,^43^.

### Zeta potential and molecular weight cut-off

The zeta potential of the prepared membranes evaluated from streaming potential and streaming current measurements is shown in Fig. 4(c). The zeta potential, which determines the antifouling performance of the membrane, was significantly increased by increasing the GO content of the PPSU matrix. A membrane prepared with a higher concentration of GO had a more negative value than the pure PPSU membrane owing to the increase in the oxygen-containing functional groups on the membrane surface. According to the results, the zeta potential values at maximum pH ~8.5 were −10 mV for PPSU, −12 mV for nanocomposite PPSU/GO 0.2 wt.%, −14 mV for PPSU/GO 0.5 wt.% and −18 mV for PPSU/GO 1.0 wt.%, respectively. As observed in the results, the pure PPSU membrane showed a less negative zeta potential than the nanocomposite membrane. Therefore, BSA did not adhere to the membrane surface, leading to the membrane being less fouled. Hence, addition of GO to the PPSU matrix helped to construct a membrane with a negatively charged surface, which led to a significant increase in the electrostatic repulsion between the membrane and proteins and support of the antifouling performance. Figure 4(d) shows the results of MWCO. MWCO is a pore characteristic of the membranes and it is related to 90% rejection of given neutral solute. As revealed in the results, the addition of GO in PPSU solution increased MWCO of the PPSU/GO nanocomposite membrane progressively from ~25 kDa to ~40 kDa. An increasing MWCO of the membrane is due to an availability of functional groups of GO, leading to increase the solution thermodynamic instability in the phase inversion process, thereby significantly increased the pore. As is known that MWCO has a linear relationship with the pore size of the membrane.

### Pure water volumetric flux and protein rejection

**Figure 5:**
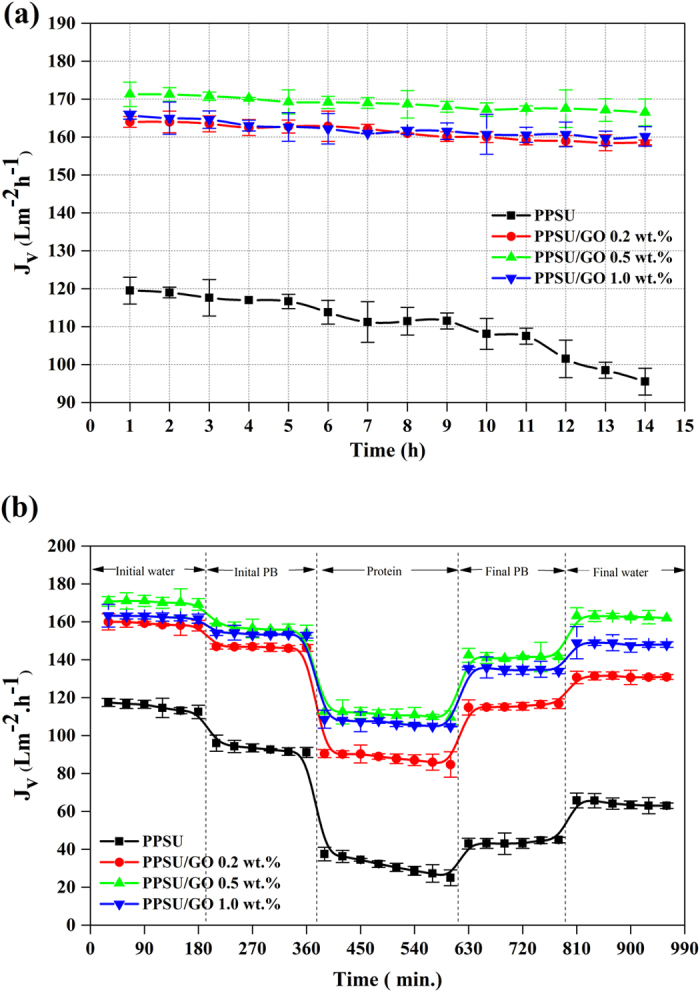
Membrane performance of PPSU, PPSU/GO 0.2 wt.%, PPSU/GO 0.5 wt.% and PPSU/GO 1.0 wt.% membranes. **(a)** Time-dependent pure water volumetric fluxes of the prepared membranes at 2 bar TMP. **(b)** Time-dependent fluxes of pure water, PB, and BSA protein and after fouled membranes.

Figure 5(a) shows the influence of GO content on membrane flux at a pressure of 2 bar. As can be clearly seen in the results, the volumetric flux of the pure PPSU membrane was 119 ± 3 Lm^−2^h^−1^. It reached 164 ± 2 Lm^−2^h^−1^ and 171 ± 3 Lm^−2^h^−1^ when 0.2 and 0.5 wt.% GO were incorporated. However, for 1.0 wt.% of GO the volumetric flux of the membrane was found to decrease, reaching 165 ± 1 Lm^−2^h^−1^. After a certain time period, the flux of the PPSU membrane declined up to 20 ± 1%, while the fluxes for the PPSU/GO nanocomposite membranes were almost constant, demonstrating that membranes prepared with GO have antifouling properties owing to the enhancement of their hydrophilicity and lower values of tortuosity of the membrane^44^. However, as the concentration of GO increased to 1.0 wt.% the decrease in the flux became more pronounced. The decrease in the flux can be explained by the increase in the entire membrane resistance, which mainly resulted from reduced surface porosity due to fouling, a greater thickness of the skin layer and suppression of the finger-like pores. Table 2 illustrates rejection of the model proteins BSA (69 kDa) and pepsin (35 kDa) by all the prepared membranes. During experiment, it was observed that protein rejection through PPSU membrane remained constant for the first 40 minutes, then rejection level increased over increasing time, meanwhile membrane flux decreased. This can be explained by the effect of fouling as well as concentration polarization caused by the electrostatic interaction between the solutions and the membrane surface charge. The rejection of both proteins BSA and pepsin were significantly enhanced with the addition of GO contents, as expected. Generally, the protein rejection is dominated by the sieving behaviour, which is determined by the membrane pore size and molecular weight of the solute, as well as the electrostatic interaction owing to membrane surface charge properties and solute isoelectric points under different pH conditions^45^. As to the size of the proteins, BSA has a larger size than pepsin, hence BSA was repelled significantly by the nanocomposite membrane^42^,^46^. As to the surface charge of the proteins at pH 7 ± 0.2, BSA (pI–4.8) shows a lower net negative charge and pepsin (pI- 1) indicates a higher net negative charge. According to Fig. 4(c), the surface zeta potential values of the membranes varied with the GO contents and reached values of −12 mV, −13 mV and −18 mV, respectively, at pH 7 ± 0.2. These values indicate a strong electrostatic repulsion between the protein molecules and the surface of the membrane; thus, the nanocomposite membrane showed a significant rejection of BSA and pepsin. In a continuation of the study of membrane fouling, protein adsorption and flux recovery ratio are discussed in the next sections.

### Antifouling property

The antifouling property of the pure PPSU and PPSU/GO nanocomposite membranes were analysed by the flux recovery, total fouling, reversible volumetric flux decline and irreversible volumetric flux decline of pure water and phosphate buffer solution after fouling by 1 g/L BSA solution as shown in Fig. 5(b). It can be observed in the results that the permeation rate of the membranes in all of the steps followed a similar trend, i.e., the volumetric flux of water as well as the phosphate buffer solution was much greater than the flux of the BSA solution. The BSA volumetric flux declined dramatically as a function of time for the pure PPSU membrane. The rate of flux decline indicated the higher fouling tendency caused by the deposition and adsorption of protein molecules on the membrane pores and surface. The PPSU/GO nanocomposite membranes retained their volumetric fluxes well at the end of protein filtration and showed the highest fluxes compared with the pure PPSU membrane. This can be explained by the effect of the GO contents, which improved membrane properties including hydrophilicity, surface charge and morphology^30^,^31^,^32^,^47^. Therefore, the nanocomposite membrane surface attracted fewer protein molecules than the pure PPSU membrane. The *J*_*v*_*RR, F*_*t*_*R, J*_*v*_*rD* and *J*_*v*_*irD* were calculated and the values are depicted in Table 2. After washing with phosphate buffer solution for the first cycle of volumetric flux, the *J*_*v*_*RR* values were 46 ± 2.1%, 78 ± 1.5%, 89 ± 4.9% and 87 ± 1.1% for the PPSU, PPSU/GO 0.2 wt.%, PPSU/GO 0.5 wt.% and PPSU/GO 1.0 wt.% membranes, respectively. The second cycle of *J*_*v*_*RR* (%) analysed by pure water volumetric flux were 55 ± 2.9%, 82 ± 1.9%,95 ± 1.4% and 91 ± 4.2% for the PPSU membrane with a GO content of 0.2 wt.%, 0.5 wt.% and 1.0 wt.%, respectively. These values indicated that the higher the flux recovery ratio, the better the antifouling properties of the membranes. The greatest antifouling performance was obtained by 0.5 wt.% of GO blended with the PPSU polymer matrix membrane. The high *J*_*v*_*RR*, in the case of the nanocomposite membrane, can be credited to the combined effects of high negative zeta potential (Fig. 4c), lower roughness and high hydrophilicity (Fig. 4a) of the membrane surface, which prevented fouling as well as the adsorption of protein molecules on the surface^48^. The introduction of GO also resulted in the changes of membrane pore sizes and thereby significantly affect membrane fouling rate. The obtained *J*_*v*_*RR, F*_*t*_*R, J*_*v*_*rD* and *J*_*v*_*irD* trend were also explained by the results of AFM images presented in Fig. 2. As shown in these images, the pure PPSU membrane had a surface with a larger area of ridge-valley structure, while the nanocomposite membrane showed a relatively smooth surface and there was found to be less of a ridge-valley structure; thus, proteins could not accumulate in the “valleys”. Therefore, the fouling possibility decreased, leading to a higher *J*_*v*_*RR*. These results demonstrated a significant role of GO in developing antifouling nanocomposite membranes and the prepared membrane can be used extensively in ultrafiltration processes.

### Protein adsorption

**Figure 6:**
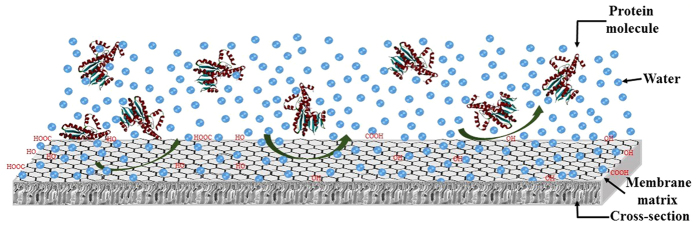
Schematic illustration of nanocomposite membrane surface and protein molecules interactions.

To evaluate the antifouling property of a membrane, a protein adsorption experiment was carried out by keeping the membrane in protein solutions for a certain time period. The adsorption results are provided in Table 2. According to the results, the adsorption percentage decreased significantly from 59.2 ± 2.5 μg/cm^2^ to 21.1 ± 2.6 μg/cm^2^ for PPSU and PPSU/GO nanocomposite membranes owing to the higher hydrophilic nature of the membrane confirmed by the water contact angle and zeta potential. Lowering protein adsorption in the case of nanocomposite membranes has been explained very briefly in earlier sections. Finally, the results revealed that PPSU/GO nanocomposite membranes had a better ability to repel the larger protein molecules (as shown in Fig. 6) and more reliable antifouling properties than the PPSU membrane and can be used in the food processing industry, pharmaceutical industry, waste water treatment, and separation of macromolecules such as bacteria, viruses, enzymes and proteins.

## Conclusion

Graphene oxide (GO) at three different concentrations (0.2, 0.5 and 1.0 wt.%) was incorporated into a polyphenylsulfone (PPSU) matrix to develop PPSU/GO nanocomposite membranes. The separation performance of the prepared membranes was evaluated by measurements of volumetric flux and rejection of BSA and pepsin as well as their antifouling properties, all as a function of the GO concentration. The results showed that the figure of merit for separation by the membrane prepared with GO showed a significant dependence on the GO concentration. The addition of GO with 0.5 wt.% resulted in a nanocomposite membrane with increased surface hydrophilicity that increased the volumetric flux from 119 ± 4 Lm^−2^h^−1^ to 171 ± 3 Lm^−2^h^−1^ with a MWCO of ~25 to ~40 kDa. The fouling resistance of the membrane was significantly improved with an optimal addition of GO (0.5 wt.%) as a result of the increase in the hydrophilicity, roughness and negative zeta potential values of the membrane surface that mitigates the protein adsorption on the membrane surface. An incorporation of GO efficiently inhibited the interaction between protein and membrane surface and thereby improved the fouling resistance by 58 ± 3%. Moreover, the membranes with GO addition showed a significant improvement in mechanical and thermal properties.

## Materials and Methods

### Materials

Polyphenylsulfone (PPSU) (Radel^®^ -R5500) was purchased from Solvay Advanced Polymer (Belgium). N-methyl-pyrrolidone (NMP) and polyvinylpyrrolidone (PVP, 10 k) were used without any further purification from Oxford Lab Chem (India). Graphene oxide (GO), bovine serum albumin (BSA), pepsin, potassium dihydrogen phosphate, disodium hydrogen phosphate dehydrate, polyethylene oxide (PEO, MW of 10 kDa), and polyethylene glycol (PEG, MW 35 kDa) were purchased from Sigma Aldrich (USA) and potassium chloride was purchased from Acros Organics (USA). Hydrochloric acid was purchased from Fisher Chemical (UK), sodium lauryl sulfate GRG from Winlab (UK) and polyethylene glycol (PEG, average MWs of 600 Da, 1 kDa, 4 kDa, 10 kDa and 30 kDa, potassium dichromate and sodium azide from Merck (Germany). Deionized water (Milli-Q) with a resistivity of 18.2 MΩ cm was used throughout the experiments (Millipore, USA).

### Preparation of PPSU/GO nanocomposites

To prepare the PPSU/GO nanocomposite membrane matrix, the PPSU and GO were first dried for 12 h at 50 °C to remove moisture content. Then, a certain amount of GO (0.2, 0.5 and 1.0 wt.%) was added to the NMP and sonicated using an digital sonifier (Branson Ultrasonics Corporation, USA) for 1 h to disperse the GO. Then the uniformly dispersed GO was mixed with the solution prepared by dissolving 17.5 wt.% PPSU and 1.0 wt.% PVP into 81.5 wt.% NMP. After stirring at 50 rpm and 70 °C for 24 h, the resulting homogeneous solution was kept until no air bubbles appeared in the solution. This was the membrane casting solution.

### Fabrication of membranes

The prepared solution of the PPSU/GO nanocomposites was cast on a clean glass plate using a casting knife film applicator (DeltaE Srl, Italy). Then, the cast film with a thickness of 150 μm was immediately immersed in a non-solvent bath of distilled water mixed with 0.5 g/L of sodium lauryl sulfate at 15 ± 2 °C to complete the phase separation. The fabricated (so-called PPSU, PPSU/GO 0.2 wt.%, PPSU/GO 0.5 wt.% and PPSU/GO 1.0 wt.%) nanocomposite membranes were preserved in deionized water containing 0.2% sodium azide to avoid microbial contamination until use.

### Membrane morphology

The cross-section morphology of the prepared membranes was examined by scanning electron microscopy (SEM; JEOL, Japan). For examination, the membrane sample was cut into pieces of various sizes and immersed in liquid nitrogen. The frozen membrane was broken and pasted onto an SEM stub with the help of conductive double-sided carbon tape. Then, the stub with the pasted samples was gold sputtered. The membrane surface topographies were analysed by atomic force microscopy (AFM) using a Nanosurf^®^ Mobile S scanning probe-optical microscope (Switzerland). For this analysis, the sample was cut into small pieces with a size of 2 mm × 2 mm and pasted onto the sample holder using adhesive tape. Then the sample holder was placed on the AFM scanner and an optical head.

### Thermal and mechanical stability measurement

The thermal stability of the membrane samples was investigated by thermogravimetric analysis (TGA; Mettler Toledo, Austria). For this test, a 10 mg sample was placed in a ceramic crucible and analysed under an inert nitrogen atmosphere (100 mL/min) under dynamic conditions between 100 °C and 650 °C at a heating rate of 10 °C/min. The mechanical properties of the membrane samples were studied using the LR5K Plus tensile test machine (Lloyd Instruments Ltd., United Kingdom). For each sample, the values of the tensile strength, breaking stress and breaking elongation were determined as the average of at least triplicate membrane samples.

### Contact angle, porosity, mean pore radius and pore tortuosity measurement

The membrane surface wettability was analysed by the contact-angle measuring instrument Attension T330 (Biolin Scientific). The measurements of all samples for the contact angle between the water and the membrane surfaces were performed using deionized water as the analysis liquid. Deionized water (3 μL) was dropped in six random locations on the dried membrane surface and the average value was reported.

The porosity (ε) of the membrane was determined by the gravimetric method, as defined in the following eq. (1) 27:


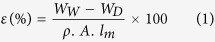


where, *W*_*W*_: weight of the wet membrane, *W*_*D*_: weight of the dry membrane, *A*: membrane effective area (m^2^), *ρ*: water density (0.998 gcm^−3^) and *l*_*m*_: membrane thickness (m).

The mean pore radius of the membranes was determined by using porosity and water flux values, and was calculated by the Guerout–Elford–Ferry eq. (2) 26,27,48:


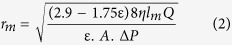


where, *r*_*m*_: mean pore radius (nm), *η*: water viscosity (8.9 × 10^−4^ Pa s), *Q*: volume of the permeated pure water per unit time (m^3^s^−1^) and Δ*P*: operation pressure (2 bar).

The tortuosity (τ) of membrane was determined by using eq. (3) 44,49:


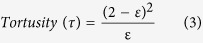


### Zeta potential measurement

The surface charge of the membrane was evaluated by zeta potential measurement using the SurPASS electro-kinetic analyser (Anton-Paar KG, Graz, Austria) based on a streaming potential and streaming current measurement. Experiments were carried out in an adjustable clamping cell and pH titration was performed from pH 2 to 9 using a solution of 0.01 mM KCl and 0.25 M HCl (acid). The resultant values of zeta potential were determined on two membrane samples for each type of membrane.

### MWCO measurement

The surface pore size of the membrane was measured in terms of MWCO using a 1 g/L neutral solution of polyethylene glycol and polyethylene oxide with different molecular weights from 600 Da to 100 kDa. The solutions were prepared individually using deionized (Milli-Q) water. Experiments were carried out on the cross-flow membrane set-up at 1 bar transmembrane pressure (TMP) at room temperature. In addition, solute concentrations of organic carbon in the feed and permeate samples were analysed by a Sievers 5310 C total organic carbon (TOC) analyser (GE Analytical Instruments). The solute rejection (R) percentage was calculated by the following eq. (4):


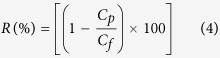


where, *C*_*p*_: concentration of PEG in the permeate and *C*_*f*_: concentration of PEG in the feed.

### Volumetric flux and protein rejection

The ultrafiltration performance of the membranes was carried out using a pressure-driven filtration CF042 cross-flow membrane module, Sterlitech Corporation (USA), where the effective membrane area was 42 cm^2^. The water permeation performance was determined by allowing deionized water (Milli-Q) to pass through the membranes using the above-mentioned cross-flow filtration system. Initially, membranes were compacted at 3 bar of TMP for 2 h to remove any remaining solvent or unreacted polymer. After compaction, values of volumetric flux at constant TMP (2 bar) were measured every 1 h for 14 h under steady-state conditions at room temperature. The flux was calculated using the following eq. (5):


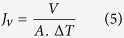


where, *J*_*v*_: volumetric flux (Lm^−2^h^−1^), *V*: volumetric flow rate of permeating water (Lh^−1^), *A*: effective surface area of the membrane (m^2^) and Δ*T*: sampling time (h).

The protein rejection experiment was performed using 1 g/L BSA or pepsin as feed solutions prepared in a phosphate buffer (0.1 M, pH 7 ± 0.2). The rejection experiments were performed at a 2 bar transmembrane pressures (TMP) for 1 h and room temperature. The protein concentrations in the feed and permeate were determined by a UV spectrophotometer (Agilent Technologies, Cary 60 UV–Vis) at a wavelength of 280 nm. The rejection value was calculated using eq. (4).

### Antifouling studies

For the antifouling studies, three cycles of experiments were carried out for each membrane sample. The antifouling property and volumetric flux recovery ratio (*J*_*v*_*RR*) of the membrane were measured using the following steps: First of all, a pure water volumetric flux was performed at 2 bar TMP for 180 min, then, initial volumetric flux of the membrane measured using 0.1 mol/L phosphate buffer (PB) under the above-mentioned condition. After this, a 1 g/L of aqueous solution of BSA as a fouling agent (pH 7.0 maintained by PB) was filtered for 240 min and the volumetric flux profile with time was recorded. After filtration of the BSA solutions, the fouled membranes were washed again using PB and the final phosphate-buffered volumetric flux was measured. Thereafter, the water volumetric flux was measured again up to 180 min for the membrane and total procedure time of 960 min.

Finally, for measuring the antifouling property of the membrane samples, the volumetric flux recovery ratio (*J*_*v*_*RR*), total fouling ratio (*F*_*t*_*R*), reversible volumetric flux decline ratio (*J*_*v*_*rD*), and irreversible volumetric flux decline ratio (*J*_*v*_*irD*) were calculated using the following eqs (6, 7, 8, 9)^27^:


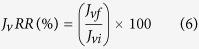



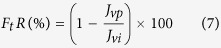



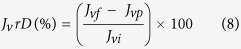



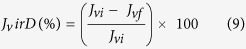


where, *J*_*vi*_: initial water or PB, *J*_*vf*_: final water or PB, and *J*_*vp*_: protein volumetric flux.

### Protein adsorption studies

Further, the antifouling property of the prepared membrane was measured by a protein adsorption test. For this measurement, BSA was used as a model protein to evaluate the anti-protein-adsorption performance. For the experiment, the membrane samples were cut into pieces with a 9 cm^2^ surface area and treated by ultra-sonication for 20 min in a phosphate-buffered solution (pH 7.0 ± 0.2) for cleaning. Then, the pre-treated membranes were kept in a phosphate-buffered BSA solution (1.0 g/L) for 24 h to attain adsorption–elution equilibrium at room temperature. After the adsorption of protein, each membrane was taken out of the solution. The initial and final concentrations of protein in the solution were determined on the basis of the absorbance wavelength. The protein concentration was measured using the Bradford micro-assay technique^27^ against a standard of pure protein solution. The apparent amounts of BSA adsorbed by the membranes were calculated using the eq. (10)^50^:


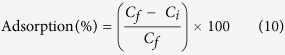


where, *C*_*i*_: initial concentration of protein solution, and *C*_*f*_: final concentration of protein solution.

## Additional Information

**How to cite this article**: Shukla, A. K. *et al*. Development of a nanocomposite ultrafiltration membrane based on polyphenylsulfone blended with graphene oxide. *Sci. Rep.* **7**, 41976; doi: 10.1038/srep41976 (2017).

**Publisher's note:** Springer Nature remains neutral with regard to jurisdictional claims in published maps and institutional affiliations.

## Figures and Tables

**Table 1:** Mean pore radius (*r*_*m*_), and tortuosity (τ) of the nanocomposite ultrafiltration membranes.

Membrane	*r*_*m*_ (nm)	τ
PPSU	8.3 ± 0.4	2.56 ± 0.21
PPSU/GO 0.2 wt.%	9.7 ± 0.3	2.14 ± 0.13
PPSU/GO 0.5 wt.%	10.6 ± 0.6	1.80 ± 0.28
PPSU/GO 1.0 wt.%	9.9 ± 0.3	2.08 ± 0.31

**Table 2:** A performance of PPSU and PPSU/GO nanocomposite ultrafiltration membranes.

Membrane	BSA rejection (%)	Pepsin rejection (%)	*J*_*v*_*RR* (%)	*F*_*t*_*R* (%)	*J*_*v*_*rD* (%)	*J*_*v*_*irD* (%)	BSA adsorbed (μg/cm^2^)
PPSU	97 ± 2	93 ± 2	55 ± 2.9	72 ± 2.1	27 ± 3.2	44 ± 2.3	59.2 ± 2.5
PPSU/GO 0.2 wt.%	95 ± 1	91 ± 2	82 ± 1.9	44 ± 1.1	26 ± 1.7	17 ± 2.8	31.0 ± 1.7
PPSU/GO 0.5 wt.%	94 ± 2	88 ± 2	95 ± 1.4	30 ± 3.3	25 ± 3.1	4 ± 1.1	25.7 ± 3.1
PPSU/GO 1.0 wt.%	95 ± 2	90 ± 1	91 ± 4.2	34 ± 2.7	25 ± 1.9	8 ± 1.5	21.1 ± 2.6

BSA and pepsin rejection (%) at a 2 bar TMP for 1 h and room temperature using 1 g/L feed solution concentration. For fouling test, the flux recovery ratio (*J*_*v*_*RR*), total fouling ratio (*F*_*t*_*R*), reversible flux decline ratio (*J*_*v*_*rD*), irreversible flux decline ratio (*J*_*v*_*irD*) for using 1 g/L BSA feed solution concentration (pH 7 ± 0.2) at 2 bar TMP for 4 h and total procedure time of 16 h at room temperature; for BSA protein adsorption (%), the 9 cm^2^ membrane surface area were kept in a phosphate-buffered BSA solution (1.0 g/L) for 24 h at room temperature.
